# Differences in gene expression between high and low tolerance rainbow trout (*Oncorhynchus mykiss*) to acute thermal stress

**DOI:** 10.1371/journal.pone.0312694

**Published:** 2025-01-08

**Authors:** Leah A. Turner, Anne A. Easton, Moira M. Ferguson, Roy G. Danzmann

**Affiliations:** 1 Department of Integrative Biology, University of Guelph, Guelph, Ontario, Canada; 2 Ontario Aquaculture Research Centre, University of Guelph, Elora, Ontario, Canada; University of Idaho, UNITED STATES OF AMERICA

## Abstract

Understanding the mechanisms that underlie the adaptive response of ectotherms to rising temperatures is key to mitigate the effects of climate change. We assessed the molecular and physiological processes that differentiate between rainbow trout (*Oncorhynchus mykiss*) with high and low tolerance to acute thermal stress. To achieve our goal, we used a critical thermal maximum trial in two strains of rainbow trout to elicit loss of equilibrium responses to identify high and low tolerance fish. We then compared the hepatic transcriptome profiles of high and low tolerance fish relative to untreated controls common to both strains to uncover patterns of differential gene expression and to gain a broad perspective on the interacting gene pathways and functional processes involved. We observed some of the classic responses to increased temperature (e.g., induction of heat shock proteins) but these responses were not the defining factors that differentiated high and low tolerance fish. Instead, high tolerance fish appeared to suppress growth-related functions, enhance certain autophagy components, better regulate neurodegenerative processes, and enhance stress-related protein synthesis, specifically spliceosomal complex activities, mRNA regulation, and protein processing through post-translational processes, relative to low tolerance fish. In contrast, low tolerance fish had higher transcript diversity and demonstrated elevated developmental, cytoskeletal, and morphogenic, as well as lipid and carbohydrate metabolic processes, relative to high tolerance fish. Our results suggest that high tolerance fish engaged in processes that supported the prevention of further damage by enhancing repair pathways, whereas low tolerance fish were more focused on replacing damaged cells and their structures.

## Introduction

Fish are one of the most taxonomically diverse vertebrates [1], inhabiting and tolerating a wide range of ecological extremes [2–4]. To thrive in these environments, fish must be able to respond adaptively to abiotic changes, both genetically and physiologically. Despite the ability of various fish taxa to withstand environmental challenges, temperature remains the major ecological factor affecting survival for many species [5–7] especially in the face of changing climate [8]. Exposure to warmer temperatures can trigger a wide range of metabolically expensive physiological changes, mediated by widespread changes in gene expression [9, 10]. Understanding the short-term physiological and longer-term evolutionary responses to rising temperatures will be key to developing policy, and mitigating the effects of climate change on fish species globally [11, 12].

Insight into the temporal nature of the thermal stress response in fishes has been gained using different methods over different experimental time scales. The earliest studies involved recording times to 50% mortality following abrupt immersion of fish into a series of water baths with increasing temperatures to obtain estimates of the species- or population-specific upper incipient lethal temperature (UILT) [13]. Alternatively, critical thermal maximum trials (CTM) have focused upon the individual-specific capabilities to withstand thermal stress where loss of equilibrium (LOE) is used as an endpoint [14]. The model CTM procedure proposes acute, continuous temperature increases (~0.3°C per minute) over approximately one hour until individual fish reach LOE [14]. Modifications to the model CTM approach, wherein temperature is raised to UILT and then held constant until LOE is reached, have also been used to examine thermal tolerance limits in fishes [15].

The rapid rates of temperature increase over a short period that characterize acute CTM trials have been criticized as not reflecting conditions experienced in the wild [16]. Thus, it has been recommended that experiments where temperature increases of 1–2°C per day, conducted over the course of days or weeks to induce a chronic temperature exposure response [16, 17] would provide insights into more adaptive thermal stress responses. Generally, longer term experiments that deem lethality as the endpoint have been termed chronic lethal methodologies [18] or incremental thermal maximum [19] studies if there are intervals of temperature stasis introduced in the temperature profiles. Findings comparing both acute and chronic methods suggest that physiological responses to either time course may be quite different [19]. Thus, studies examining both acute and chronic timescales will allow a greater understanding of how species respond to temperature increases in both the short- and longer-term.

Acute studies have demonstrated that one of the first lines of defense against thermal stress in fishes is the highly conserved heat shock response (HSR) [20–22], which is induced when the thermal stress is sufficient to trigger wide-scale protein denaturation. The accumulation of denatured proteins in the cell results in the detachment of the heat shock transcription factor from the heat shock element, allowing for transcription and translation of heat shock proteins (HSPs) [23]. HSPs such as *hsp70*, *hsp30*, and *hsp90* act as chaperones that help to refold mature proteins that are beginning to denature [24] and are upregulated as part of the HSR [10, 25–29]. Indeed, CTM studies have demonstrated that upregulation of transcripts associated with gene ontology (GO) terms linked to protein folding and stability occurs in the earliest stages of heat shock [30, 31]. Studies at the chronic level have also noted upregulation of heat shock proteins within 24 hours of the fish first experiencing an increase in temperature [10, 32]. To support the increased demand for protein synthesis that accompanies the HSR, protein translation machinery genes are also upregulated in the early stages of thermal stress [10, 31, 33]. Whether this upregulation in protein synthesis continues at longer time scales is unclear, with some authors identifying genes and pathways associated with protein synthesis being upregulated [10, 34], while others have observed downregulation in these pathways [35].

Aside from the gene expression changes associated with the HSR, thermal stress also changes gene expression relating to cellular turnover and remodelling, which is required because of denaturation of transmembrane proteins. This denaturation can trigger dysfunction in plasma membrane structure and function [34, 36]. At both acute and chronic time scales, synthesis of cytoskeletal genes, such as actin and tubulin [37], and changes to (un)saturated fatty acid levels are necessary to repair damaged cellular membranes and to reduce membrane fluidity in response to increases in temperature [36, 38]. If cells and proteins have been damaged beyond repair, they must be cleared away, requiring an upregulation of apoptosis and ubiquitination genes, respectively [25, 37, 39–42]. Cleared damaged cells must be replaced, resulting in upregulation of DNA replication and cell cycle-associated genes at both acute [31] and chronic [10, 32, 34, 43] timescales. Proteins are replaced by an upregulation in cellular translation machinery in both acute and chronic studies [10, 33].

The many physiological and genomic adjustments required to cope with thermal stress culminate in a dramatic increase in energy consumption in the short-term, both directly and indirectly. The routine metabolic rate rises as a direct result of an increase in temperature [17, 44], and as an indirect result of the increased costs of protein synthesis [45, 46] as described above. Fish must therefore both reduce energy use, and/or increase ATP synthesis to cope with the stress of increased temperatures, both in the short- and longer-term. At acute CTM time scales, fish appear to reduce energy usage by downregulating genes classified under the DNA repair and replication GO terms [31], and by downregulating transcription- and translation-related pathways, such as aryl-hydrocarbon-receptor signalling [29] and ribosome production [30]. At chronic timescales, energy savings are derived from downregulation of biosynthesis pathways, such as those of lipids, cholesterol [32], and steroid hormones [34]. Energy savings are also achieved by a reduction in standard metabolic rate as the fish acclimates to the higher temperature [5, 47]. Producing sufficient energy to supply increased demand during stress requires coordination of several organs, arguably the most important of which is the liver. For example, in thermally stressed rainbow trout, the liver increases glucose production, which in turn enters the blood supply to fuel other organs’ energy production [48], and acts as a storehouse for glycogen, a key anaerobic substrate [49–51]. Furthermore, the liver is innervated by the sympathetic and parasympathetic nerve fibres and thus plays an important role in sensing and rapidly responding to physiological and pathological changes in the organism (reviewed in [52, 53]).

A large body of work has identified and focused on changes to metabolic rate as a key driver and predictor of the response of ectotherms to climate change (reviewed in [54]). However, other factors as discussed above may also be driving the response to thermal stress, but these are less studied. Previous studies have also largely focused on interrogating a pre-selected suite of genes with qPCR [31, 40, 55–57] or have used RNA-Seq [31, 37, 58] to better understand the broader transcriptomic responses of thermally stressed, relative to control fish. However, few studies have used RNA-Seq on multiple strains to get not only a whole-transcriptomic approach, but one that is more generalizable to a given species [59, 60], nor to our knowledge have previous studies compared the responses of high and low tolerance fish to thermal stress. Thus, more comprehensive studies are required to fully recognize the extent of the impacts of changing temperatures on these organisms. Specifically, it is important to identify individual variation in how conspecifics respond to thermal stress given that such variation is how individuals adjust in the short term and how populations respond at evolutionary time scales.

We studied the genomic and physiological basis of thermal tolerance in the liver from two strains of rainbow trout to better understand the impact of warming on acclimation capacity [61] and thus, the ability to respond to a warming climate [9]. We focused on whole liver (including associated nervous, vascular, connective tissues) due to its key role in metabolic processes and its ability to respond rapidly to thermal stress due to innervation by the autonomic nervous system. Our first goal was to determine the large-scale, liver-specific transcriptomic responses to acute thermal stress during CTM trials with a time scale of 16–18 hours by comparing the gene expression profiles of fish with low and high acute thermal tolerance from two strains to establish which patterns of gene expression are associated with these levels of thermal tolerance. Our second goal was to identify differentially expressed genes (DEG) shared across strains, which could serve as important signatures in the adaptive response to thermal stress in the species. Doing this provides a comparative framework for future interspecific comparisons. Our third goal was to gain a broad perspective on the functional processes associated with high thermal tolerance across both strains based on gene ontology (GO) analysis. To gain a finer perspective on the functional processes involved across both strains, our fourth goal was to identify (a) functional pathways of genes associated with high thermal tolerance and (b) upstream regulators that are predicted to be either inhibited or activated in high tolerance fish due to their interactions with other DEG based on Ingenuity Pathway Analysis (IPA) [62]. By using broad-scale whole-genome transcriptomic analysis, we aim to generate novel insights into the pathways and mechanisms through which rainbow trout induce an acute adaptive response to thermal stress.

## Materials and methods

All experiments were carried out at the Ontario Aquaculture Research Centre (OARC; Elora, ON, Canada) and at the Hagen Aqualab (University of Guelph, ON, Canada) in strict accordance with the Canadian Council for Animal Care guidelines, under a University of Guelph Animal Care Committee approval, protocol #3550.

### Crosses

On 8 November 2017, 34 half-sib families from the Lyndon strain (Lyndon Fish Hatcheries; New Dundee, ON, Canada) were created by crossing 17 males and 17 females in a step-chain design, with each parent mated twice. However, only one half-sib family from one of the females survived. This resulted in 27 families being available for the experiment produced from 15 females (with 3 females only contributing to one family), and 16 males (with 5 males contributing to only 1 family).

On 10 October 2018, 31 families from the Alma strain were produced by crossing 16 females to 16 males from OARC using a similar half-sib step-chain design. Although 32 families should have been produced using this design, one of the females was inadvertently used in only 1 half-sib cross. Consequently, one of the males was only represented as a singleton cross. Also, a pair of half-sib families from one of the females failed to develop, as did one half-sib family from another cross, resulting in only 28 families being reared. Therefore, the final crosses for the experiment included two females out of 15 being involved as a singleton parent, and four males out of 16 being included as singleton males. All remaining parents were involved in two half-sib crosses.

### Fish rearing at OARC

Embryos were reared in vertical incubating racks at 8°C until approximately 1 week post-hatching, at which time the fish were moved to 0.5m family tanks. An average of 190 fish/family from the two strains were transferred to larger (0.7m) family tanks around 45 days post-fertilization (dpf). Fish were fed daily and housed at 8°C with constant aeration *via* an air stone to maintain normoxic conditions (~9–10 mg L^-1^ O_2_) and periodic monitoring of nitrates and nitrites.

On 8 February 2018 (13 weeks pf), 6 fish from each Lyndon family (except for one family where only 5 fish were transferred) were randomly placed into one of six pools, for a total of 161 fish per pool. On 2 and 3 May 2019 (29 weeks pf), the procedure was repeated with the Alma strain, but with 4 fish per family randomly placed into one of eight pools, one family having only 3 fish transferred due to poor survivorship, and other families having 5 fish transferred to produce 112 fish per pool. Lyndon fish have intrinsically higher growth rates than Alma fish, and the increased densities in Lyndon pools were expected to result in similar growth rates in the two strains.

On 19 July 2018, fish from the Lyndon strain were anaesthetized in a bath of tricaine methane sulfonate (MS-222; Sigma-Aldrich, Oakville, ON, Canada) and elastomer tagged by pool using Visible Implant Elastomers (VIE; Northwest Maritime Technologies, WA, USA). Similarly, on 11 June 2019, the 8 Alma pools were identified by elastomer tags in preparation for transport to Hagen Aqualab facility at the University of Guelph.

### Critical thermal maximum (CTM) trials

To determine transcriptomic responses to acute thermal stress and to compare the patterns of differential gene expression between low and high tolerance fish of both strains we used a discontinuous critical thermal maximum (CTM) trial protocol, as described below.

### Transportation to Hagen Aqualab

On 10 August 2018, fish from the Lyndon strain were transported to the Hagen Aqualab. Upon arrival, 17–19 fish from each of the six Lyndon pools were selected at random and placed into one of eight 315 litre experimental tanks (61 x 244 x 28 cm), to ensure that each replicate tank had representation from all pools reared at OARC. The fish were held in 10°C water for a week, and then the water temperature was increased to 12°C during the second week prior to the CTM trial. The fish were supplied with constant aeration of the water (~9–10 mg L^-1^ O_2_) and monitored for nitrate and nitrite levels. Prior to the trial, eight fish were removed (1 per tank) to serve as a control experimental group (C) leaving 875 fish to be subjected to the CTM trial.

On 28 June 2019, the Alma strain fish were transported to Hagen Aqualab. Fourteen fish from each of the eight Alma pools were randomly assigned to each of the eight experimental Aqualab tanks. The fish were reared for two weeks prior to the CTM trial as described in the above paragraph. Prior to the trial, eight fish were removed (1 per tank) to serve as a control experimental group (C) leaving 886 fish to be subjected to the CTM trial. Although strains were sampled in different years, fish were of similar ages and developmental stages at the time of their respective trials, minimizing the effects of sampling year on fish performance.

### CTM trial protocol

At the end of the two-week acclimation period, food was withheld for 72 hours. The CTM trials were conducted on 22 August 2018 (288 dpf; Lyndon strain) and 10 July 2019 (274 dpf; Alma strain) using loss of equilibrium (LOE) as the phenotypic score endpoint for each fish. LOE is defined as the complete loss of ability of a fish to swim upright [14]. Upon reaching a the LOE endpoint, each fish was euthanized with an overdose of MS-222. We removed whole livers from fish selected as control, low and high tolerance and placed this organ into RNA*later* (Invitrogen, Burlington, ON, Canada) and stored at 4°C. Wet mass (g) and fork length (FL, mm) were also measured on these fish.

We used a glass rod to prod fish that had lost equilibrium to further ensure they could not regain equilibrium, if they were able to move in an inverted position. Once a fish reached LOE, it was removed from its replicate tank and euthanized in a bath of MS-222 as described above. The time taken to reach LOE and temperature at LOE were recorded, and the fish were identified to their OARC pool by their elastomer tag with a UV light and were frozen (-20°C) shortly thereafter. Wet mass and length measurements were later made on these fish.

**Fig 1:**
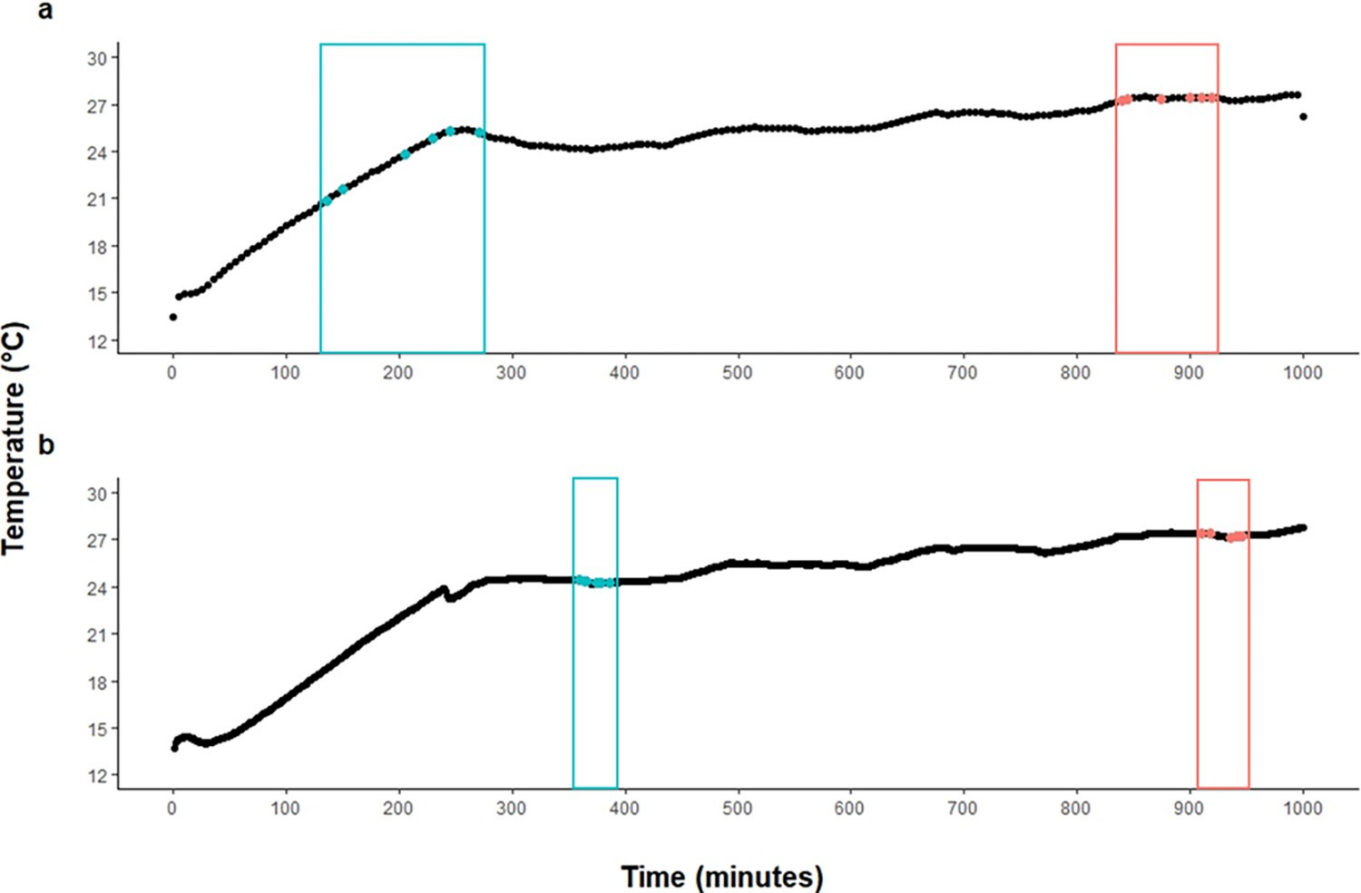
Critical thermal maximum (CTM) temperature ramping protocols used for the a) Lyndon and b) Alma rainbow trout thermal tolerance trials. Points at which low tolerance fish were sampled are indicated in blue, while points at which high tolerance fish were sampled are indicated in salmon.

The discontinuous CTM trial was started by increasing the temperature from 12°C to 24°C at a rate of ~3.4°C hour^-1^ for the Lyndon trial (Fig 1A) and at a rate of ~2.7°C hour^-1^ for the Alma trial (Fig 1B). These differences in the rate of temperature increase were not intentional but occurred due to accidental differential water flow rates through the heat exchanger in the two separate trials. This may have affected the speed with which the fish reached LOE, but since we sampled the fish at the ends of the distribution (i.e., early and late fish to reach LOE), we were still able to identify the fish with the lowest and highest thermal tolerance within each strain. Upon reaching 24°C, the temperature was maintained for 3 hours, after which the temperature was increased to 25°C over a 1-hour period and maintained at that level for a 3-hour interval. Temperature steps were programmed to increase in this manner up to 28°C, but less than 1% of the fish remained at the end of the 27°C interval. Temperatures were continuously monitored at source inflow (1 minute intervals) using a computer-controlled Argus electronic system (Argus Controls, Surrey, BC, Canada), which also controlled the heat exchanger temperature profile.

Six fish that reached LOE early in each trial were designated as the low tolerance experimental group (L; Table 1). Sampling this group was straightforward given the slow rate at which fish lost equilibrium at the lower temperatures. The high tolerance experimental group (H) was sampled from the final 10% of the fish to reach LOE. Extra fish were sampled at both ends of the distribution to allow for exclusion of very small or very large fish from either strain, because of the potential effect of mass on thermal tolerance (reviewed in [63]).The fish we selected as H and L experimental groups in the Lyndon strain (Fig 1A) differed by approximately 680 minutes to CTM, and those in the Alma strain differed by approximately 560 minutes in their time to CTM (Fig 1B). The H and L fish within strains did not differ significantly in mass according to an unpaired t-test using the R Project for Statistical Computing (RRID:SCR_001905) version 4.0.0 [64] (Table 1).

**Table 1:** Summary of critical thermal maximum (CTM) trials by strain and by thermal tolerance.

Strain	Thermal tolerance	Sample size (N)	CTM (°C; mean ± SEM)	Time from onset of experiment to CTM (minutes; mean ± SEM)	Mass (g; mean ± SEM)	Median rank	Range of ranks
Lyndon	Low	6	23.64 ± 0.38	235.65 ± 21.90	75.95 ± 8.35	17.5	7–26
	High	6	26.77 ± 0.02	916.17 ± 11.79	63.42 ± 9.82	823	767–840
Alma	Low	6	23.81 ± 0.01	371.33 ± 4.21	38.77 ± 2.24	11.5	4–23
	High	6	26.84 ± 1.59E-15	932.50 ± 6.11	43.52 ± 3.14	779	736–790

Fish mass within the low and high tolerance fish of each strain were not significantly different from one another according to an unpaired t-test. The last two columns refer to the rank order in which fish attained LOE during the experiment.

### RNA extraction and illumina sequencing

Whole livers were removed from RNA*later* and blotted with KimWipes to remove salts before extracting total RNA using the QIAGEN RNeasy Mini Kit (QIAGEN, Toronto, ON, Canada). The RNase-Free DNase Set (QIAGEN, Toronto, ON, Canada) was used to remove any genomic DNA (gDNA) contamination. RNA samples were checked for potential degradation and/or gDNA contamination using a bleach agarose gel made using TAE buffer, as described in Aranda et al. [65]. Briefly, samples were heated to 85°C for 10 minutes and chilled on ice for 3 minutes prior to loading onto the gel to resolve any secondary structures that may have formed. All RNA extracts were assessed for quality using the Nanodrop 8000 Spectrophotometer (RRID:SCR_018600), and any samples that did not have a 260/280 ratio ≥ 2.0 were re-extracted. A subset of the samples was run on the Agilent 4150 TapeStation System (RRID:SCR_019394) at the Genomics Centre (GC) within the Advanced Analysis Centre (AAC) at the University of Guelph for additional quality assurance.

Samples were assessed for quality and quantity at the GC using the Agilent 2100 Bioanalyzer Instrument (RRID:SCR_019389) and Bioanalyzer RNA 6000 Nano assay (Agilent Technologies, Mississauga, ON, Canada). Average RNA integrity number for the samples was 9.26 and ranged from 7.2–10. RNA samples were then submitted to Génome Québec (GQ; Montreal, QC, Canada) for sample processing. GQ created mRNA libraries from the total RNA, using the NEBNext Ultra II Directional RNA Library Prep Kit for Illumina (New England Biolabs, Ipswich, MA, USA), according to the manufacturer’s instructions. GQ then sequenced RNA-seq libraries on an Illumina NovaSeq 6000 Sequencing System (RRID:SCR_016387) as 100bp paired-end reads according to the manufacturer’s protocol. RNA-Seq reads were submitted to the NCBI SRA library (RRID:SCR_004891) under project ID PRJNA209213 which may be accessed at www.ncbi.nlm.nih.gov/sra/?term=PRJNA209213. The low and high tolerance and control samples for the Alma strain can be accessed under sequential numbers SAMN43369603 –SAMN43369620 (18 samples), and similarly SAMN43369621 –SAMN43369638 (18 samples) for the Lyndon strain, or run numbers SRX25830506 –SRX25830541 inclusive for both strains combined. Sample specific *.fastq downloads may be obtained from ebi.ac.uk/ena/browser/home using the above SRX accession numbers or using the SRA download toolkit from NCBI. A version of the above protocol is available at dx.doi.org/10.17504/protocols.io.5jyl8pxerg2w/v1.

### Data analysis

#### RNA-Seq data

Adapter sequences and barcodes were removed from the sequences by GQ, but subsequent data preparation (quality control and trimming) was conducted using the default parameters of the CLC Genomics Workbench (RRID:SCR_011853) version 21.0.4 (QIAGEN, Aarhus, Denmark). After filtering and trimming, certain paired reads were uncoupled and occurred as singletons/orphans, but this number was low (< 0.001% of all reads in either strain). RNA-Seq analysis was also conducted using the CLC Genomics Workbench again according to default parameters, with the exception that total counts used to generate values were obtained by counting paired reads as two, and orphans as one, and for unannotated transcripts RPKM values were calculated using the length of the gene. The rainbow trout reference genome assembly OmykA_1.1 (GCF_013265732.2), available from NCBI Genome (RRID:SCR_002474), was used to map sequence reads.

#### Differential gene expression

To address our first goal of characterizing the liver-specific transcriptomic responses to increased temperature, we first assessed the general patterns in gene expression among the L, H and C experimental groups in each of the two strains. We then calculated normalized counts (i.e., transcript reads per kilobase per million mapped reads; TPM) for each fish and used these as input for a single principal component analysis (PCA) across both strains. We used the *PCA()*, with units centred and scaled using scale.unit = TRUE in FactorMineR (v. 2.4) [66]. Eigenvalues were extracted using the get_eigenvalues() command, and the PCA was visualized using fviz_pca_ind() in factoextra (v. 1.0.7) [67]. Five dimensions were retained in the final PCA; 84.3% of the variance explained therein, according to the Eigenvalues.

We then identified differentially expressed genes (DEG) between pairs of experimental groups (H vs. C, L vs. C and H vs. L) within each strain. The DEG analysis was done with CLC Genomics Workbench using default parameters, including ‘filter on average gene expression for FDR correction.’ All comparisons between the three pairs of experimental groups were analyzed as input from the experimental metadata table, within each strain. Significance cut-offs for gene expression analysis were made using a false discovery rate (FDR) ≤ 0.01.

To address our second goal of identifying DEG shared between strains, the DEG for a pair of experimental groups within a strain were ordered from highest to lowest gene expression level using the FDR P < 0.01 cutoff. We then determined the proportion DEG shared within an experimental group across strains for each comparison type (i.e., H vs. C, L vs. C, H vs. L). Finally, we determined the proportion of DEG within a strain that were upregulated in each of experimental groups within a comparison (e.g., those with significant expression in both the H and C groups in the H vs. C comparison). The number of DEG unique to an experimental group within a comparison type and within a strain and those shared between strains was illustrated with Venn diagrams.

### Identifying gene function through gene ontology (GO) analysis

To address our third goal of identifying the functions of DEG, we first characterized the broad-scale functional categories (i.e., Gene Ontology (GO) terms; RRID:SCR_002811) for the significant DEG identified that were shared between strains for a given comparison type. The protein FASTA (RRID:SCR_011819) sequences (USDA_OmykA_1.1) of significant genes in each pair of experimental groups were first aligned to the eggNOG-mapper (RRID:SCR_021165) version 5.0 database [68], and genes not identified using this method were further queried using *blastp* alignments (NCBI BLAST; RRID:SCR_004870; see further descriptions in the next paragraph). The output file from the eggNOG analysis was used to obtain GO terms directly for most of the query genes. For genes that did not have GO terms identified using the eggNOG database, the Pfam (RRID:SCR_004726) motifs [69] identified within the genes by eggNOG were further searched using the GODM database [70]. To search the GO terms associated with each Pfam motif, a custom VB script (doi.org/10.5281/zenodo.13620354) was made that would assign Pfam accession IDs to each Pfam motif, and then match them with their respective biological process (BP), cellular component (CC), and molecular function (MF) GO terms from the GODM database. The completed lists of GO terms from both eggNOG and GODM databases were appended together and submitted to the Animal Database GO Terms Classification Counter (https://agbase.arizona.edu/cgi-bin/tools/goslimviewer_select.pl) tool in the USDA AgBase repository [71] to search against the Protein Information Resource (PIR; RRID:SCR_008229) [72] to obtain all the PIR GO slims for each gene and its associated GO terms. To arrange and order all the output files produced by the AgBase repository, a custom VB script (doi.org/10.5281/zenodo.13620072) was written to report the gene counts for each fish group across all three comparison types (H vs. C, L vs. C, H vs. L) associated with each unique GO slim PIR term.

Differences in the total counts and percentages of genes within each GO-slim category were determined for each pair of experimental groups. The percent counts were then used to rank all GO slim terms from highest to lowest values. Thus, the highest positive value in each comparison represents the GO slim term that differed in the greatest proportion in the first group and the lowest negative value represents the GO slim term with the greatest differences in the second group. The grouped pairwise gene counts across all GO slim categories were then submitted as comma-delimited files to a custom VB script (doi.org/10.5281/zenodo.13133983) that calculated which of the multiple GO terms were significantly overrepresented in either experimental group via a backward elimination heterogeneity G-test procedure. The data was structured such that positive differences represented terms overrepresented in the H compared to the L group in the H vs L comparison, and H group in the H vs. C, and the L group in the L vs. C comparisons, while negative terms represented terms overrepresented in the C (in H vs. C), C (in L vs. C), and L group (in H vs. L) comparisons. Finally, to further our knowledge of the physiological factors that enhance the performance of high tolerance fish, we performed a comparative analysis of the GO terms that were both shared and different between H fish in the H vs. C and H vs. L experimental groups.

### Identifying gene function via pathways and upstream regulatory genes

To address goal 4, we first identified canonical pathways and major upstream regulatory genes and their functions affecting thermal tolerance based upon predictive mammalian gene interaction networks as determined by the QIAGEN Ingenuity Pathways Knowledge Base (IPA) [62] (RRID:SCR_008117). The IPA software is based upon information in humans, mice, and rats, and as such, has limitations for direct comparisons with fish genes. Nonetheless, many gene functions are conserved among vertebrate species, making it beneficial to explore how the gene functional networks identified in these model mammalian species may help to elucidate the underlying basis of the response to thermal stress in rainbow trout. As with the GO analysis, we focused the IPA analysis on the subset of DEG shared across strains as determined from the three pairwise comparisons of experimental groups. We then used this information to identify pathways that are either enhanced or repressed in H fish compared to L fish.

To convert the rainbow trout DEG into their orthologous mammalian designations we used the rainbow trout protein sequences to search the NCBI and eggNOG mapper databases (v2.1.2 to 2.1.4) for their mammalian equivalents. This was first done by protein-protein aligning the rainbow trout genes to eggNOG database and searching for the respective mammalian (i.e., mouse (*Mus musculus*) and human (*Homo sapiens*) orthologues. For genes not identified by the eggNOG database, *blastp* alignments BLAST (BLASTP; RRID:SCR_001010) against the general NCBI database (ncbi.nlm.nih.gov) searches were conducted to find human orthologues to the rainbow trout genes. Default *blastp* parameters were used, except for the following: general parameters: maximum target sequences = 50, expect threshold = 1.0E-03, word size = 3; scoring parameters: matrix = BLOSUM45, gap costs = existence: 14, extension: 2; filters and masking: low complexity regions were filtered. These parameters mirrored the eggNOG default settings. The human/mouse orthologues were chosen by selecting the accession number with the lowest e-value and highest bit-score, % identity, % query coverage, and BLAST results with e-values ≥ 0.001 were not used. In cases where two or three orthologues were equally likely, the functional description of the original rainbow trout gene was used as a guide. The gene was no longer considered in instances where several different matches (> 3) were equally possible. The resulting list of selected protein accession numbers for the human/mouse orthologues were then matched against their corresponding Ensembl (RRID:SCR_002344) or GeneID (RRID:SCR_021639) from NCBI, and these, along with the fold-change and FDR-adjusted p-value for each gene obtained from the CLC Genomics Workbench analysis were used as input for the IPA analysis. A maximum of 8000 genes can be analyzed in IPA in any single analysis; thus, we adopted a threshold of FDR P < 0.01 across all three pairwise experimental analyses to ensure the number of genes analyzed did not exceed this maximum.

During assembly of the rainbow trout datasets and their conversion into mapped IPA gene IDs, we observed that multiple trout genes were being mapped to identical mammalian gene IDs. This indicated that our data set contains a large complement of duplicated genes. Although most of the duplicated copies showed consistent directions in gene expression between two experimental groups, some inconsistencies were present. In instances where duplicates are present, IPA selects the duplicate copy with the highest gene expression level (either positive or negative) for further analysis. In most instances, this selection is representative of the direction of expression for all duplicated copies of that gene. There were exceptions, however, where the additive total expression of the duplicated copies showed opposite expression to the selected copy. We chose not to remove these genes from the data set as this may have led to removal of key genes in regulatory pathways.

To generate lists of IPA canonical pathways and to identify upstream regulators, an expression-based core analysis was undertaken for each of the H vs. C, L vs. C, and H vs. L comparisons. Within each comparison, the expression level and p-value for each DEG shared between strains was used as input into IPA. Program default settings were used except that “node types” pertaining to biologic drug, various endogenous and exogenous chemicals, and mature microRNA and microRNA focal points were not considered as we were primarily interested in the gene set entered and their interactions. Other knowledge base information criteria were retained as default settings. The canonical pathway results were further filtered by omitting any directional pathway (i.e., z-score > 0 or < 0) with -log(p-values) < 1.3, as they represent minor interacting gene sets. However, neutral pathways (i.e., z-scores of NA or 0), were retained if they possessed -log(p-values) > 1.3, as such pathways represent major sets of interacting genes that have bidirectional influences on the thermal response. The lists of upstream regulators generated by IPA were examined and we focused only on those with significant log_2_-fold expression changes in our study. Therefore, any gene that did not show significant expression differences between two experimental groups was not considered nor reported. Additionally, following IPA recommendations, only regulatory genes with a z-score exceeding ± 2.0 were categorized as major. While a complete list of these major upstream regulators with their gene expression differences for each pair of experimental groups is presented in S7 File, the presentation and discussion of findings is focused on the H vs. L comparison.

Like the GO analysis previously described, we compared pathways in H fish from the H vs. L comparison to their expression in the H vs. C comparison group to determine which pathways appear to be more enhanced or suppressed in H fish during the acute thermal challenge. In other words, most pathways that are elevated in expression in H fish in comparison to L fish are also elevated in expression compared to C fish. However, some notable exceptions to this pattern were evident and represent pathways that appear to be more enhanced or repressed.

We then performed a functional effects analysis in IPA to identify biological functions enhanced in both H and L fish. This was done using all the shared FDR P < 0.01 genes in the Alma and Lyndon strains. From this, we obtained a list of the top ranked group-specific functions (z-score > ± 1.6) within H and L fish based upon the gene interaction effects of all the genes in the analysis. This list of top functions allowed us to identify broader categories of functions which were the focus of our discussion. In addition, we were also interested in examining the functions associated with the top-ranked upstream regulators in both H and L fish. This was of interest as it provides information on the range of functions that may be associated with these top regulators and facilitates comparisons between the two groups.

To further characterize the functions that distinguish H vs L fish, we compiled a listing of functions associated with top ten ranked upstream regulators found within both H and L fish. This ranking was obtained using both the z-score rank and expression level ranks for genes that matched IPA predictions with respect to their expected expression level profiles within the dataset (i.e., genes expected to be upregulated and that were observed to have greater expression levels in H fish, or downregulated and were observed to have greater expression levels in L fish). To survey the associated functions for these genes we obtained the listing of associated functions from both the IPA ‘BioProfiler’ and IPA ‘Functional Effects’ Knowledge databases. To query the ‘Functional Effects’ database, we filtered association reports for each gene using only the ‘Functions’ and ‘Others’ datasets and omitting the ‘Diseases’ subset in the output settings. This was accomplished by searching the Knowledge Base using > 70 keyword wildcard terms (e.g., cardio*, carbo*, development*, growth*, lipo*, etc; see NOTES worksheet in S10 File for complete listing). Since IPA emphasizes pathways related to human cancers, we omitted those terms from the subcategory listings to obtain a filtered list more directly related to basic physiological processes. The associations define whether increased expression of a regulatory gene ‘affects’, ‘increases’, or ‘decreases’ the function of the physiological process.

During our analyses we observed that there were multiple instances of mismatches for upstream regulators wherein IPA predicted expression levels for a regulator did not match observed expression levels. Therefore, it was of interest to re-examine the gene-function associations by querying the IPA ‘functional effects’ listing of genes against the gene expression levels in the dataset. The trout gene-functions associations were assessed using a custom script (doi.org/10.5281/zenodo.13620590) that generates a Functional Effects Group (FE_g_) score. Briefly, this assigns a group score associated with whether genes in a group increase, decrease, or affect a given function when expression for those genes are enhanced, and then tests for differences in the proportions of genes in these three categories between the two groups. We first examined the match between upstream regulators in both H and L fish (N = 412). The same analysis was then performed using all the shared significant (FDR P < 0.01) genes in the dataset (N = 4650).

## Results

### Characteristics of the RNA-Seq dataset

The overall characteristics of the processed RNA-Seq data were very similar between the two strains. Total reads ranged from 56,854,754–94,177,754 (median = 72,498,421), and 54,648,998–102,403,700 (median = 73,890,125) reads in the Alma and Lyndon strains, respectively (S1 File). Orphaned and discarded reads from the read trimming resulted in very low percentage values (~1.0E-06%) in both strains. Unmapped reads were also very similar in both strains (median = 2.44% and 2.29% of total processed reads in the Alma and Lyndon strains, respectively). Overall, the average number of unmapped reads were higher in the H and L groups (mean = 3.16% and 2.57% in the Alma and Lyndon strain, respectively), compared to the control group (mean = 1.79% and 2.05% in the Alma and Lyndon strains, respectively; S1 File). The percentage of unmapped reads was also very similar across all individuals within both strains, except for one of the low tolerance fish from the Alma strain (Alma-Low-02; S1 File). This low tolerance fish had 8.10% unmapped reads compared to levels of around 2.5% for the other fish in the study.

### Differential gene expression

**Fig 2:**
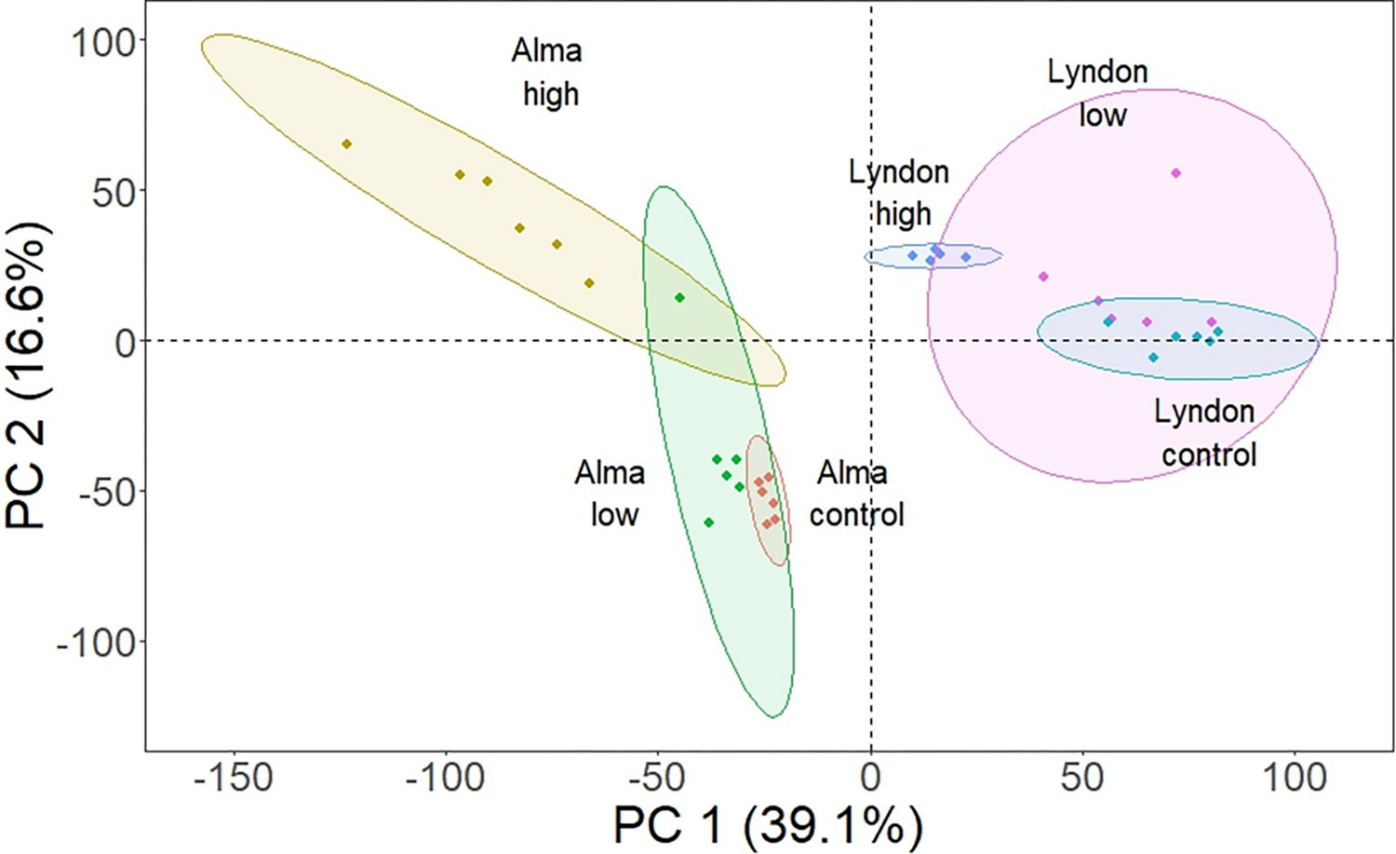
Variation in liver gene expression profiles (RPKM) of significant genes (FDR ≤ 0.01) in the Lyndon and Alma strains of rainbow trout based on PCA. PC1 and PC2 represent the combinations of genes that explain 39.1 and 16.6% of the gene expression differences between the comparison groups, respectively.

Gene expression patterns among the H, L, and C experimental groups differed in both the Lyndon and Alma (Fig 2) strains according to the PCA of normalized counts of DEG. The three groups showed mostly non-overlapping distributions on one of the PC axes in each strain apart from a single L fish in the Alma strain that was aligned with the H group.

**Fig 3:**
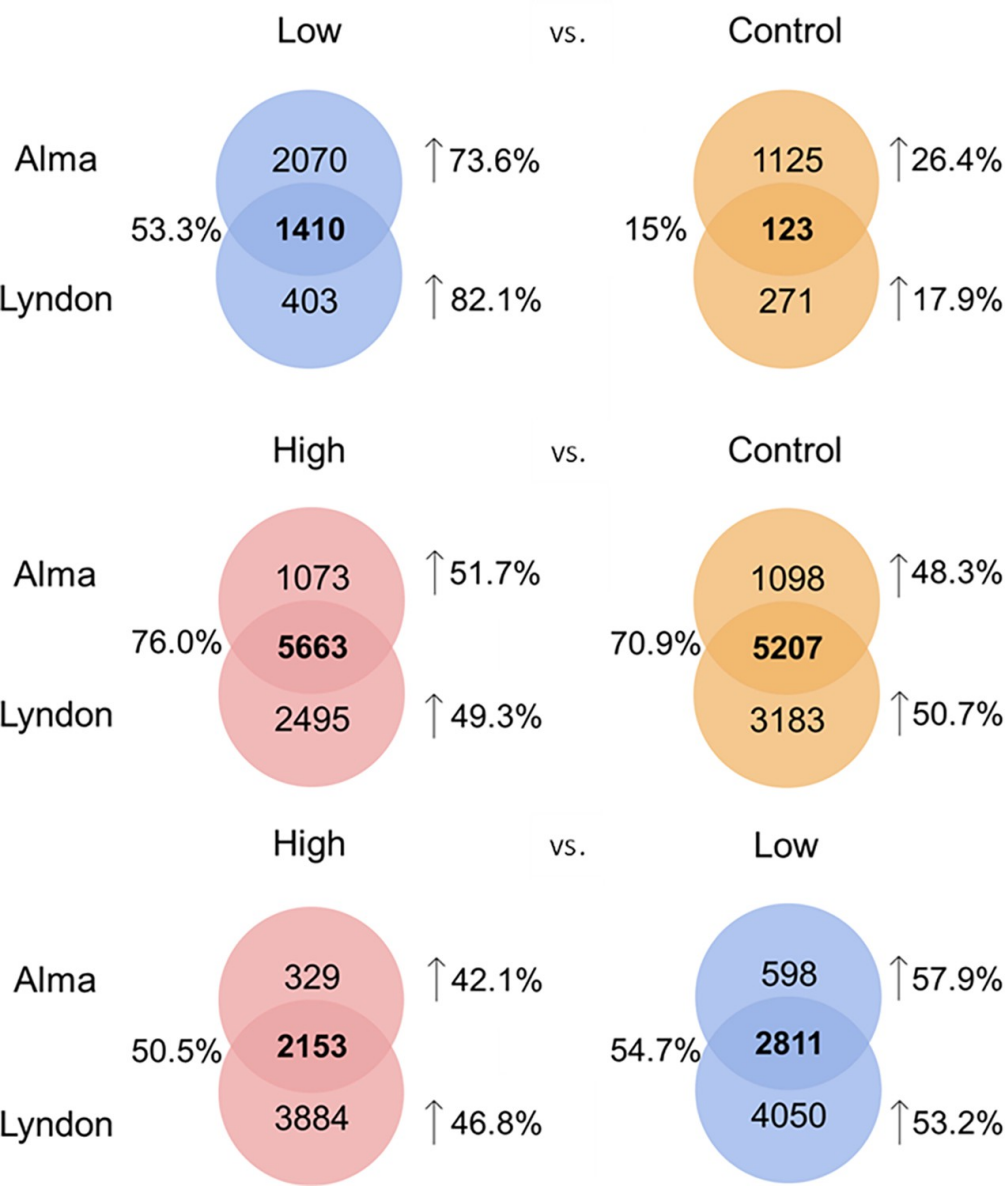
Venn diagram showing the distribution of differentially expressed genes (DEG) between experimental groups within and between strains. Bolded numbers indicate the number of shared DEG between the strains within a given comparison. Arrows next to percentages indicate the proportion of upregulated genes unique to each experimental group within a strain, while the percentages without arrows indicate the proportion of genes within an experimental group that are shared between the two strains.

The number of significant DEG varied between pairs of experimental groups and strains (Fig 3; S2–S4 Files). Of all comparisons among experimental groups, the lowest number of DEG occurred between the L and C groups. This was consistent in both strains, where the L and C comparison in the Alma strain showed greater numbers than those of the Lyndon strain (Alma: 4728; Lyndon: 2207). This suggests that the L and C groups have similar gene expression patterns. Within strains, a much greater proportion of DEG were upregulated in the L group (Alma: 73.6%; Lyndon: 82.1%) compared to the C group. The proportion of shared DEG across strains was higher for the L group (53.3%) than the C group (15%). The low degree of sharing between strains in the C groups suggests patterns of gene expression vary between strains under control conditions but are more similar when fish are exposed to increased temperatures. The most highly represented genes in the L fish included several *heat shock proteins* (*hsps*), while the C fish upregulated *ferritin*, *cytochrome P450 7A1*, and *A-kinase anchor protein SHPKAP-like*, among other genes (Table 2).

**Table 2:** Top 10 unique up- and downregulated genes in low tolerance relative to control fish, at FDR ≤ 0.01.

Gene ID	Description	Average log_2_ fold change
Low tolerance		
LOC110523814	heat shock protein 30-like (15)	13.612
LOC110525787	protein unc-45 homolog B	12.190
LOC110523813	heat shock protein 30 (2)	12.120
LOC118938279	heat shock 70 kDa protein (5)	11.864
LOC118938284	heat shock 70 kDa protein-like (7)	11.791
LOC110522488	heat shock protein HSP 90-alpha 1 (2)	11.510
hspa1b	heat shock protein family A (Hsp70) member 1B	11.353
hsp70a	heat shock protein 70a	11.244
LOC110497859	heat shock 70 kDa protein 4L	11.231
hsp70b	heat shock protein 70b	10.972
Control		
LOC110515900	ferritin, middle subunit	-8.507
LOC110530204	cytochrome P450 7A1	-6.272
LOC110503320	A-kinase anchor protein SPHKAP-like	-5.341
LOC110536311	hexokinase-4	-5.101
LOC110528221	25-hydroxyvitamin D-1 alpha hydroxylase, mitochondrial	-3.977
LOC110525277	arrestin domain-containing protein 3	-3.916
npas4l	neuronal PAS domain protein 4 like	-3.532
LOC110513818	GSK-3-binding protein	-3.219
LOC110520396	acyl-CoA-binding protein	-3.160
LOC110532702	Indian hedgehog protein	-3.075

Ranking was generated by taking the average log_2_ fold change for each shared gene. Numbers in brackets indicate the total number of duplicate copies identified for a given gene. Genes were identified by cross-referencing the gene ID with the protein accession number using the USDA_OmykA_1.1 genome assembly for rainbow trout, and any ‘uncharacterized’ genes were omitted from the list. In the case of duplicate gene copies, the gene ID refers to the copy with the strongest log_2_ fold change. The full list is available in worksheets a & d, S4 File.

In contrast to the L vs. C comparison, the H vs. C comparison detected much greater numbers of DEG in both strains with Lyndon being higher (Alma: 13,041; Lyndon: 16,548) (Fig 3). Unlike the L vs. C comparison, similar proportions of DEG were upregulated in the H and C groups in both strains. Moreover, the proportion of DEG shared between strains was similar across groups (H: 76%; C: 70.9%). However, as with L fish, H fish included *hsps* among their most highly upregulated genes, while they downregulated *seizure protein 6 homolog*, *snail family zinc finger 1b*, and *glycoprotein hormones alpha chain 1* (i.e., top upregulated genes in C fish; Table 3).

**Table 3:** Top 10 unique upregulated (high tolerance fish) and downregulated (control fish) genes at FDR ≤ 0.01.

Gene ID	Description	Average log_2_ fold change
High tolerance		
LOC110523814	heat shock protein 30-like (16)	15.540
LOC118938281	heat shock 70 kDa protein (5)	15.346
LOC110522488	heat shock protein HSP 90-alpha 1 (2)	14.198
LOC110525787	protein unc-45 homolog B	14.176
hspa1b	heat shock protein family A (Hsp70) member 1B	14.166
LOC110523813	heat shock protein 30 (2)	14.030
hsp70a	heat shock protein 70a	13.976
LOC110497859	heat shock 70 kDa protein 4L (2)	13.813
LOC110529844	heat shock protein HSP 90-alpha	12.889
LOC118938284	heat shock 70 kDa protein-like (8)	12.433
	Control	
LOC110504052	seizure protein 6 homolog	-9.128
snai1b	snail family zinc finger 1b	-8.542
LOC110505305	glycoprotein hormones alpha chain 1	-8.344
LOC110538699	Ig mu chain C region membrane-bound form-like	-7.840
LOC110507838	tumor necrosis factor receptor superfamily member 19	-7.643
LOC110528221	25-hydroxyvitamin D-1 alpha hydroxylase, mitochondrial (2)	-7.555
LOC110493598	zinc finger and BTB domain-containing protein 45	-7.394
leap-2b	liver-expressed antimicrobial peptide 2B	-7.299
LOC110538142	plexin domain-containing protein 1-like	-7.229
LOC110501325	junctional adhesion molecule B	-7.197

Ranking was generated by taking the average log_2_ fold change for each shared gene. Numbers in brackets indicate the total number of duplicate copies identified for a given gene. Genes were identified by cross-referencing the gene ID with the protein accession number using the USDA_OmykA_1.1 genome assembly for rainbow trout, and any ‘uncharacterized’ genes were omitted from the list. In the case of duplicate gene copies, the gene ID refers to the copy with the strongest log_2_ fold change. Full list is available in worksheets g & j, S4 File.

The H vs. L comparison also revealed greater numbers of DEG than the L vs. C comparison (Fig 3). Like the H vs. C comparison, Lyndon had greater numbers of DEG between groups than Alma (Alma: 5,891; Lyndon: 12,898). In addition, the proportion of upregulated DEG was similar in the H and L groups in both strains (Alma and Lyndon had 42.1% and 46.8% of DEG in the H group, respectively, and 57.9% and 53.2% of DEG in the L group, respectively). The proportion of shared genes in both groups were also very similar (H: 50.5%; L: 54.7%). The most highly represented genes in the H group were *small integral membrane protein 18* and *18-like*, and *hepatocyte growth factor activator*, while the L fish had *seizure protein 6 homolog*, *ST8 alpha-N-acetyl-neurminide alpha-2*,*8-sialytransferase* 2 among their top upregulated genes (Table 4). A complete description of the all the significant genes can be found in S4 File.

**Table 4:** Top 10 unique upregulated genes in high and low tolerance fish at FDR ≤ 0.01.

Gene ID	Description	Average log_2_ fold change
High tolerance		
LOC118948980	small integral membrane protein 18-like	9.154
LOC110531794	hepatocyte growth factor activator	7.417
LOC110525162	small integral protein 18	7.058
LOC110514559	β-1,3-galactosyl-O-glycosyl-glycoprotein β-1,6-N-acetylglucosaminyltransferase 3-like	6.548
LOC110506486	synaptotagmin-5	6.410
LOC110530204	cytochrome P450 7A1	6.154
LOC100136187	cathelicidin antimicrobial peptide	6.013
tdgf1	teratocarcinoma-derived growth factor 1	5.950
LOC110489950	zinc transporter ZIP12	5.528
LOC110500102	ankyrin repeat domain-containing protein 16-like	5.457
Low tolerance		
LOC110504052	seizure protein 6 homolog	-8.183
st8sia2	ST8 alpha-N-acetyl-neuraminide alpha-2,8-sialyltransferase 2	-7.486
LOC110507838	tumor necrosis factor receptor superfamily member 19	-7.291
c1qtnf6a	C1q and TNF related 6a	-6.505
rab9b	RAB9B, member RAS oncogene family	-6.394
si:ch211-266k8.4	carabin	-6.028
LOC110529956	adiponectin-like	-6.011
LOC110507706	septin-4	-5.999
foxn4	forkhead box N4	-5.897
LOC110501325	junctional adhesion molecule B	-5.812

Ranking was generated by taking the average log_2_ fold change for each shared gene. Genes were identified by cross-referencing the Gene ID with the protein accession number using the USDA_OmykA_1.1 genome assembly for rainbow trout, and any ‘uncharacterized’ genes were omitted from the list. Full list is available in worksheets m & p, S4 File.

### Identifying gene function through gene ontology (GO) analysis

There were large differences in the counts of significant GO terms detected across the three pairwise comparisons. While there were a substantial number of GO terms detected across all three major GO terms categories in the H vs. C and H vs. L comparisons, this was not apparent for the L vs. C comparison. The L and C groups showed a limited number of significant differences in the counts of biological process (BP) GO PIR-slim terms, while no differences were detected in the cellular component (CC) and molecular function (MF) categories (worksheets a-c, S5 File). Only 11 biological process (BP) GO slim terms showed significantly different counts between groups and six of these were higher in L fish, while the other five were lower in L fish. Collectively, the BP terms enriched in the L fish were involved with cellular localization, organelle organization, the transport of substances and molecules, and protein metabolic process, while terms pertaining to cellular proliferation and metabolic process categories were more repressed.

The H vs. C comparison detected 281 significant GO PIR-slim terms, of which 147 (52.3%) were from the BP category and of these 61 (41%) were enriched in the H fish (worksheet d, S5 File). The enriched BP terms in the H fish included processes associated with protein metabolism, folding, modification and transport. Additionally, BP categories enriched in the H fish included organization of organelles. Almost one third of the total number of GO slim terms (86, 30.6%) were from the CC category, of which 28 were enriched in H fish (worksheet e, S5 File). These included components related to transcriptional and translational processes, such as spliceosomal complex, ribosome, preribosome, polysome, ribonucleoprotein complex, RNA polymerase complex, and stress and degradation processes (e.g., endoplasmic reticulum, ubiquitin ligase complex, proteasome complex, I-KappaB/NF-kappaB complex). Similarly, the 15 significant GO slim terms enriched in the H fish from the MF category were associated with transcription and translation (worksheet f, S5 File). In contrast, BP repressed terms in H fish were more related to general metabolic processes (e.g., oxidation-reduction processes, lipid metabolic process, carbohydrate metabolic process), cytoskeletal structuring (e.g., cell adhesion, tissue remodeling), growth, DNA repair, and immune system processes. CC terms in H fish were also repressed for numerous cytoskeletal component terms, immune function terms, and structures related to transcriptional and cellular division component (e.g., transcription factor complex, kinetochore, chromosome, cell division site). Most of the repressed MF terms in C fish were related to metabolic processes (e.g., lipid, carboxylic acid, carbohydrate, vitamin binding), and signaling.

The H vs. L comparison revealed the greatest number of significant GO terms of the three comparisons. Of the 290 significant GO slim terms, the majority (55.2%) were BP, of which 56 were enriched in the H fish relative to the L fish (worksheet g, S5 File). These BP terms included several metabolic process, transport, localization, and organization related terms, as well as terms related to protein salvage and degradation and translational processes. In contrast, the BP terms repressed in H fish included carbohydrate and lipid metabolic process, lipid transport, and production of new cells. As with the H vs. C comparison, CC accounted for almost a third (30.7%) of significant GO terms, with 29 enriched in the H fish (worksheet h, S5 File). These CC terms included structural component terms related to organelles, gene expression and post-translational modifications, in addition to RNA degradation. In contrast, the H fish were repressed for CC terms including cellular membranes and cytoskeleton and cellular replication. Lastly, MF only accounted for 13 (14.1%) repressed terms in the H fish of the H vs. L comparison, (worksheet i, S5 File) but H fish were enriched for MF processes associated with translational and protein regulatory processes, protein and macromolecular turnover (e.g., hydrolase activity, ubiquitin-like protein transferase activity, peptidase activity), and were repressed for MF terms more related to energy and metabolism regulation (e.g., lipid binding, carboxylic acid binding, carbohydrate binding), and signaling (e.g., signaling receptor activity, molecular transducer activity). Overall, H fish repressed a greater number of GO terms across all three categories (66.2%) compared to L fish.

### Comparisons of GO terms represented in high tolerance fish

Examination of the GO terms in H fish in the H vs C and H vs L comparisons revealed GO terms that appear enhanced and suppressed in H fish. Ten BP GO terms pertaining to cell cycle, cell replication, and growth were suppressed in H fish from the H vs. L comparison compared to the H vs. C comparison, while terms such as cellular component disassembly, cellular respiration and oxidative phosphorylation were enhanced (worksheet r, S5 File). The only CC term enhanced in H fish was mitochondrion. Repressed terms included centrosome and ubiquitin ligase complex, and those related to transcriptional and gene expression functions. MF terms related to processing (hydrolase activity, peptidase activity) were enhanced in H fish, whereas terms such as transporter activity, ion binding, and chromatin binding were repressed (worksheet r, S5 File). A complete list of the terms shared and unique in H fish across the H vs. L and H vs. C comparisons is given in worksheets j-q, S5 File, and the associated GO term assignments to all significant genes detected in the study are provided in worksheets s & t, S5 File.

### Duplicated genes

Many of the DEG in the IPA analysis were identified as duplicated gene copies (S6 File). Of the 9402 mapped DEG shared between strains for the H vs. C comparison, 5002 had at least one duplicate copy. These 5002 duplicates represented 2166 unique gene IDs (worksheet e, S6 File). After IPA selection of the gene copy with the greatest expression level, 107 of the 2166 unique gene IDs (4.9%) varied in the direction of expression between experimental groups across strains. This mismatch in expression between strains represented 1.6% of all the unique mapped DEG between the H and C experimental groups. For the L vs. C comparison, 534 of the 1400 mapped shared orthologous gene IDs entered in the “core analysis” module were identified as duplicates (worksheet j, S6 File). This duplicate set of 534 genes corresponded to 235 unique gene IDs, and only one of these showed a mismatch in expression between strains. Finally, for the H vs. L comparison, 1717 of the 4650 mapped DEG shared across strains were duplicates and were assigned to 790 unique gene IDs. A total of 25 genes showed different patterns of expression (mismatches) between strains representing 3.2% of the duplicates found, and 0.5% of all the H vs. L genes surveyed (worksheet o, S6 File). In summary, the IPA analyses were based on 6566, 1101 and 3723 mapped gene IDs for the H vs. C, L vs. C and H vs. L comparisons, respectively.

### Ingenuity pathway analysis: Canonical pathways

Like the DEG analyses, the H vs. C comparison had more significant pathways compared to the H vs. L comparison (S7 File). In the H vs. C comparison, the two strains shared 308 canonical pathways, 105 of which were enhanced, and 203 of which were repressed in the H group (i.e., enhanced in C group; worksheet a, S7 File). Two of the pathways detected (coronavirus replication and coronavirus pathogenesis) were not considered further due to limited relevance to the current study. Additionally, 58 neutral pathways were evident across the H and C groups (worksheet b, S7 File). Graphical summaries of key processes and genes indicated elevated expression of features such as unfolded protein response, transport of protein, and autophagy in the H group while many developmental processes such as development of head, development of vasculature, development of body axis, and development of body trunk were suppressed in the H group (worksheets a & b, S8 File). In addition, processes such as cardiogenesis, fibrogenesis, vasculogenesis, and central nervous system diseases were suppressed in the H group.

In the L vs. C comparison, 197 significant pathways were shared between the Lyndon and Alma strains (worksheet e, S7 File). Of these, 181 were enhanced in the L group, and 16 pathways were enhanced in the C group. Relative to control fish, L fish upregulated canonical pathways implicated in cellular proliferation, growth, differentiation, and death, along with multiple signaling pathways (worksheets c & d, S8 File). Control fish upregulated carbohydrate, lipid and fatty acid metabolism as evidenced by the high ranking of PPAR signaling, PPARα/RXRα and PXR/RXR activation pathways, as well as having associated effects on transcriptional/translational, cell cycle and apoptotic pathways (i.e., Sumoylation Pathway, PTEN Signaling, Apoptosis Signaling). However, graphical overviews of the major processes within L vs. C fish do not highlight these processes, given the large number of pathways detected in L fish. Most of the processes highlighted in L fish relate to enhancement of development of body trunk, cardiogenesis, NRF2-mediated oxidative stress response, autophagy, unfolded protein response, quantity of leukocytes, lymphocytes, and blood cells, and transport of protein. The most enhanced pathway in L fish (cardiac hypertrophy signaling (enhanced) supports this summary.

The Lyndon and Alma strains shared 216 canonical pathways in the H vs. L comparison (worksheet i, S7 File). Of these, two were coronavirus-related pathways and were not considered for lack of relevance to the current study. Of the remaining 214 pathways, 149 were repressed and 65 were enhanced in the H group, relative to the L group. The graphical overview of the processes suggests suppression of pathways involved in vasculogenesis, formation of cellular protrusions, microtubule dynamics, angiogenesis, quantity of cells, and development of body axis in H fish (worksheets e & f, S8 File). Conversely, pathways related to stress, disease, and tissue dysregulation (e.g., renal lesion, early onset neurological disorder) were enhanced in the H fish. Two of the pathways not highlighted in the graphical summaries, but that remained consistent with H vs. L observations in terms of a high ranking were unfolded protein response and tRNA charging. In fact, tRNA charging was ranked first in both comparisons involving H fish, and unfolded protein response was ranked second in the H vs. C and 3^rd^ in H vs. L pairwise comparisons (worksheets a & i, S7 File). Those enhanced in L, relative to H, fish included metabolism of xenobiotics and lipids, as well as pathways involved with the cardiovascular system, cellular replication, and responses to cellular stress. The two most enhanced pathways in L fish were pulmonary fibrosis idiopathic signaling pathway and cell cycle control of chromosomal replication.

Analysis of the enhancement or suppression of pathways present in H fish relative to L fish, based upon their expression in the H vs C fish (S9 File), revealed a greater number of pathways that were suppressed in H fish (N = 37) compared to those enhanced (N = 22). Most notable in terms of enhancement was spliceosomal cycle which ranked second in H fish. Inhibition of matrix metalloproteases and remodelling of epithelial adherens junctions were also enhanced in H fish and are related pathways which can regulate epithelial to mesenchyme transitions (EMT) in vertebrates (reviewed in [73]). In addition, the SUMOylation pathway post-translational modification process was greatly enhanced in H fish. Conversely, several pathways related to cholesterol biosynthesis and phosphoinositide signaling were suppressed in H fish, as well as leukotriene biosynthesis, kinetochore metaphase signaling pathway, and sirtuin signaling pathway.

### Ingenuity pathway analysis: Upstream regulators

IPA analysis of the H and L groups identified 1,658 upstream regulatory genes based upon the gene interaction networks present. Most of these regulators (74.8%) were predicted to be present solely based upon the IPA Knowledge Base interactions, while 418 regulators (25.2%) also had significant expression level differences associated with predicted IPA regulatory states. The number of identified regulatory genes was greater in the L group compared to the H group. Significant differences in expression were detected for 157 genes in H fish, and 255 genes in L fish (see NOTES and m worksheets in S7 File for details).

When considering only the major regulatory genes, thirty (19.1%) of the genes within H fish were identified as major regulators (z-score ≥ 2.0), while 51 (20.0%) (z-score ≤ -2.0) were identified in L fish. Since IPA makes predictions as to whether a regulatory gene will be activated or inhibited in relation to the focal (H) group, we could assess whether the predicted regulatory states matched observed expression levels. For the 81 major regulators (z-score ± 2.0 threshold) detected, 32 had mismatches in expression relative to IPA predictions. In other words, an H gene with enhanced expression was predicted to be inhibited, while an L gene (i.e., with suppressed expression in the H group) was predicted to be activated. From this total, 12 (40%) and 37 (73%) upstream regulators matched IPA expectations, within H and L fish, respectively, regarding their predicted activation states (worksheets k-m, S7 File).

Examination of the top 10 ranked major H and L upstream regulators based on either average expression levels or z-scores across both strains indicated a greater variation in L group regulators (worksheets k & l, S7 File). This is based on those regulators with expression levels matching IPA expectations of being activated or inhibited. Given the lower number of significant upstream regulators detected in H fish (12 in total) compared to L fish, the rankings between highest expression levels and highest z-score rankings for the H regulators were essentially identical. In H fish, the gene *spdef* had the highest expression level ranking, while *nupr1* had the highest z-score rank. Because of the larger array of significant upstream regulators (37 genes) in L fish, a total list 17 genes contributed to the top 10 ranked genes across both categories. In L fish, three genes (*ckap2l*, *jag1*, and *ntrk2*) had top 10 ranks for both elevated expression and z-score levels with 7 regulators comprising the additional top ranking positions within either the expression level or z-score level group. *Adipoq* had the highest expression level ranking, while *erbb2* had the highest z-score ranking for L fish.

### Ingenuity pathway analysis: Functional effects in top-ranked regulators

When biological functions were assigned to the top upstream regulators in L (17 genes) and H (12 genes) fish (S10 File), it was evident that there was a large overrepresentation of terms (9685) in L compared to H fish (1431 terms; worksheets e & f, S10 File). This suggests that the genes within the L group have broad ranging influences on functional effects. The top three ranked genes with the greatest number of functions ascribed in H group were: *nupr1* (194 terms); *klf6* (194 terms), and *nfe2l1* (174 terms). The top three ranked genes in L fish were: *e2f1* (1624 terms); *erbb2* (1381 terms), and *ppara* (1023 terms). The average number of terms in the 12 H genes (mean = 119.5; range: 25–194) was much lower than in 17 L genes (mean = 571.2; range: 35–1624), again suggesting that the functional responses within H fish are more restricted.

### Ingenuity pathway analysis: Functional effects in H vs. L fish using all significant genes

A total of 62 functional effects (z ± 1.6 threshold) were detected across both H an L fish, with a greater number (N = 35) in L fish (S11 File). It was also evident that multiple functions within each group were related. For example, seven functions related to neurological processes were evident in H fish, whereas only three were evident in L fish. Similarly, 19 functions related to enhancement of development, growth, and morphogenesis were elevated in L fish, whereas only two functions related to these terms were evident in H fish. Both terms in H fish suggested suppression of growth rather than enhancement. Placement of the functional effects identified into broader categories indicated that many were related to: a) transcription and translation; b) cell cycle; c) neural regulation; d) autophagy; e) aspects of energy metabolism; and f) inflammation. Given the complexity of many of the details involved with the latter two topics, they will be presented in future publications.

When the association between observed gene expression levels and their predicted effects on biological functions were re-examined (FE_g_ analysis in S11 File), it revealed a strong match to IPA predictions in L fish, but a poor match in H fish. A greater correspondence to IPA expectations was obtained when considering the analysis based upon all genes in the dataset. Within L fish, the ‘all gene’ analysis revealed that the function related to survival/viability of cancer cell lines were not significant between H and L fish, whereas other functions matched IPA predictions for L fish (see worksheet d, S11 File). In H fish, when the data was reanalyzed, only 10 out of 27 functions matched IPA expectations using either the ‘upstream regulator’ or ‘all genes’ analysis. Most notably, many neurological-related categories did not match expectations. They were either predicted to be more elevated in L fish or showed no differences between H and L fish (worksheets c & d, S11 File). These observations prompted us to investigate in more detail the functions of genes associated with each of the major functional groupings described in the previous paragraph. Details on these individual gene functions are outlined in the Discussion.

## Discussion

We assessed the common transcriptomic responses to acute thermal stress in two strains of rainbow trout with the goal of better understanding the physiological outcomes arising from gene expression changes driving thermal tolerance. We observed some of the classic responses to increased temperature, such as the unfolded protein response (UPR) and induction of the *heat shock proteins* (*hsps*), but these were not the defining factors that differentiated high (H) and low (L) tolerance fish. Instead, differences in tolerance between H and L fish appeared to involve divergent responses in several primary areas. First, relative to L fish, H fish selectively upregulated a lower diversity of raw transcripts while making variant transcripts and stabilizing and destabilizing mRNAs as needed, allowing them to respond to thermal stress more efficiently. Second, while H fish decreased cellular proliferation and preserved cellular integrity, L fish increased progression through the cell cycle, potentially accruing cellular damage. Third, H fish regulated neurotransmitter release and mitigated oxidative stress to avoid neuronal death, whereas L fish succumbed to dysregulation of neurotransmission and ensuing excitotoxicity. Lastly, while both H and L fish increased their autophagic activity in response to thermal stress, H fish limited damage to cellular structures by increasing aggrephagy and microphagy activities and invested in lysosome repair, while L fish replaced lysosomes by upregulating lysosomal biogenesis in response to thermal stress.

### Transcription and translation

Higher thermal tolerance was associated with the selective upregulation of genes related to the cellular stress response, as indicated by H fishes’ enhanced transcription, translation, and splicing activities (worksheets a & b, S12 File). Relative to L fish, H fish increased transcription by upregulating key activators (including *cyclin-dependent kinase 9; cdk9*) [74], and increased the mRNA elongation machinery by upregulating key genes, including the rate-limiting *eukaryotic translation initiation factor 4e* (*eif4e*) [75]. As well, core translational components such as several ribosomal RNA proteins (RPs) and ribosome biogenesis/assembly genes, including *nucleolin* (*ncl*) [76, 77], *urb1 ribosome biogenesis homologue* [78, 79] and *upstream binding transcription factor* (*ubtf*) [80], were upregulated in H fish. In addition, tRNA charging levels which reflect protein synthesis rates [81] were also significantly enhanced in H fish, and indeed, *tRNA charging* was the top-ranked IPA canonical pathway reported for H fish. The upregulation of transcription and translation was targeted at a lower diversity of genes, as evidenced by lower numbers of GO terms and enhanced IPA pathways relative to the L fish. This suggests that synthesizing a greater quantity of fewer genes may be an efficient strategy to cope with thermal stress.

The maintenance of lower transcript diversity in H fish may have been achieved by upregulating pathways that exert more control over which genes are expressed, and by selectively stabilizing and destabilizing mRNA in response to changing conditions within the cell [82, 83] (S4 File; worksheets a & c, S12 File). One translation machinery subcomponent whose expression was elevated in H fish was *eukaryotic translation initiation factor 2 subunit 1* (*eif2s1*), whose phosphorylation in response to endoplasmic reticulum (ER) stress suppresses translation of most genes, with the exception of genes required for the unfolded protein response (UPR) [84–89]. In turn, *eif2s1* upregulates *activating transcription factor 4* (*atf4*), whose protein activates several important UPR-related genes, such as those involving the antioxidant response, amino acid metabolism, and autophagy (reviewed in [88]). Another key transcription-regulating pathway enhanced in H fish was the IPA SUMOylation pathway. While SUMOylation is a post-translational modification (PTM) that can result in either transcriptional activation or repression, Sumo proteins recruitment by *death domain-associated protein* (*daxx*; upregulated in H fish) results in transcriptional repression [90–92]. Additionally, *daxx* can bind with transcription factors, histones, and chromatin-related proteins, further influencing expression of target genes [93]. Thus, the targeted gene expression stemming from *eif2s1* and *atf4*, along with Sumo-*daxx* transcriptional control, is associated with increased thermal tolerance as observed in H fish relative to L fish.

The regulation and (de)stabilizing of mRNA processing and the process of alternate splicing may have also contributed to localized control of gene expression in more thermally tolerant fish. H fish regulated mRNA (de)stabilization to allow for continued expression and translation of otherwise unstable genes [94] by upregulating *poly(a) binding protein cytoplasmic 1* (*pabpc1;* worksheets a-c, S12 File) [83, 95–97], and by upregulating genes whose products can act as stabilizers or promote mRNA decay as needed by the cell such as *heterogenous nuclear ribonucleoprotein d* (*hnrnpd*) [98–100] and *synaptogamin binding cytoplasmic RNA interacting protein* (*syncrip*) [101–103]. H fish also upregulated genes typically associated with mRNA decay, such as several *YTH N*^*6*^*-methyladenosine (m*^*6*^*A) RNA-binding protein (ythdf*), *ccr4-not transcription complex subunit* (*cnot*) family genes [82, 104], and *5’-3’ exoribonucleases 1* and *2* (*xrn1*, *2*) [95, 105]. Rapid decay of mRNA limits its translation, thus providing the cell with a means for selective gene expression in response to thermal stress [106]. Alternate splicing also supported selective gene expression in response to thermal stress by producing more stable proteins. Indeed, components of the *spliceosomal cycle* IPA pathway were enhanced in H fish (second top-ranked IPA canonical pathway; S7 File), and numerous *U1*, *U2*, *U4*, *U5*, and *U6 ribonucleoprotein* genes were largely more highly expressed in H fish, as were several splicing factor genes and one of the three *exon junction complex (ejc)* genes (i.e, *mago homolog* (*magoh*), as well as the key regulator of the *ejc* (i.e., *RNA binding motif protein 8a* (*rbm8a*); worksheet b, S12 File). Upregulation of spliceosomal genes has also been observed in fishes exposed to heat and cold stressors at both acute and chronic timescales [33, 107–110]. Interestingly, much like the L fish in this study, the thermally intolerant nototheniid species *Pagothenia borchgrevinki* failed to upregulate spliceosomal components in response to chronic thermal stress [43]. Spliceosomal activity may have been supported by SUMOylation, which may be a potent regulator of spliceosomal activity [111]. Thus, an enhancement of the *spliceosomal complex* IPA pathway, supported by enhanced SUMOylation, might have helped the H fish produce a more thermally stable suite of genes during the thermal stress.

### Cell cycle

Increased thermal tolerance was associated with reduced expression of many cell cycle regulatory genes (both enhancers and repressors) in combination with increased expression of a smaller subset of genes that repress the cell cycle (worksheet d, S12 File). For example, H fish had enhanced expression for CDK5 (inhibits neuronal cell proliferation [112], *e2f transcription factor 6 (e2f6;* inhibits progression from G_1_ to S phase; reviewed in [113], *wee1* (inhibits progression through the G2 checkpoint) [31, 114], and *Krüppel-like factor 6* (*klf6*; prevents damaged cells from reproducing) [115]. This suggests the importance of halting the cell cycle and conserving energy through reduced gene expression as a component of increased thermal tolerance. In contrast, L fish showed enhanced expression for most major regulators that facilitate cell cycle progression, such as numerous additional cyclin dependent kinases (e.g., *cyclin-dependent kinase 1*;*cdk1*) [116, 117], cyclins (e.g., *cyclin b1; ccnb1*) [118, 119]. and *cytoskeleton associated protein 2 like* (*ckap2l*) [120]. L fish also upregulated cell division cycle, E2F transcription factors, and structural component genes. Not halting the cell cycle can result in DNA damage, which can have downstream effects if cells accruing this damage are allowed to replicate [121] leading to reduced thermal tolerance.

In addition to halting the cell cycle, H fish may have protected cytoskeletal integrity by downregulating *matrix metalloproteases* (*mmps*). Mmp proteins not only degrade structural proteins in the extracellular matrix (ECM) [122, 123], but are also important for regulating architecture of tissues [124]. For example, Mmp proteins participate in repair of wounds and of the basement membrane of epithelial cells [125], thus creating space for new cells to proliferate [124, 125]. Mmps also play key roles in cellular growth, proliferation, and migration [126–128]. While L fish upregulated several *mmps*, H fish enhanced the *inhibition of matrix metalloproteases* IPA pathway, thus preventing these activities and preserving cellular integrity. Given that the cost of protein synthesis is high, and the replacement of cells comes at a high metabolic cost [129, 130], replacing cells is detrimental to energetic balance during periods of thermal stress, which could account for the higher thermal tolerance of H fish.

### Neuroregulation

One of the features of high thermal tolerance in H fish was the apparent suppression of glutamate- and γ-aminobutyric acid (GABA)-mediated neurotransmission, thus mitigating excess neurotransmission and glutamate toxicity (worksheets e & f, S12 File). Under non-stressed conditions, glutamate and GABA transmission are involved in controlling the balance between excitatory and inhibitory activity in the brain, respectively [131, 132]. Under stress conditions, this balance is disrupted, leading to excess glutamate signaling [133] largely mediated by an excess *glutamate ionotropic NMDA receptor* (*grin*) expression. This is further compounded by the fact that under stress conditions, the *GABA receptor subunits alpha* and *beta* (upregulated in L fish) switch from their function as mediators of inhibitory neurotransmission [132] to facilitators of glutamate transmission [134]. Unchecked receptor-mediated glutamate neurotransmission has been associated with nervous system hyperactivity (reviewed in [135]), leading to neuron death (i.e., excitotoxicity; reviewed in [136]), particularly when mediated through *grin2b* [137] (upregulated in L fish). Given that H fish have lower levels of NMDA and GABA alpha/beta receptor activity, they can more effectively regulate extracellular glutamate levels.

Modulation of genes involved with glutamate neurotransmitter release was also observed in H fish leading to the inhibition or dampening of evoked neurotransmitter release (worksheets e & f, S12 File). One such means by which this was achieved was through the SNARE complex (among the enhanced IPA pathways in H fish; S7 File), which is a critical mediator of synaptic vesicle formation [138, 139]. H fish also upregulated genes associated with the SNARE complex, such as *complexin 3* (*cplx3*) and *cplx4* which can slow synaptic vesicle formation [140]. Furthermore, *cplx3* and *cplx4* cooperate with *synaptotagmins* (*syt*) [141], such as *syt11* (4^th^ highest ranked differentially expressed gene in H fish), to limit neurotransmission [142, 143], which can be greatly elevated during increased ER stress [144]. Thus, increased expression of *cplx3*/*4* and *syt11* in H fish may have helped to dampen the neurotransmission levels that accompany the thermal stress response. H fish further dampened neurotransmission by suppressing cholesterol biosynthesis (i.e., IPA Cholesterol Biosynthesis Pathway I, II, and III). Lower levels of membrane cholesterol correlate with lower levels of neurotransmission in general [145], which may have helped the H fish reduce the release of extracellular glutamate, thus minimizing its neurotoxic effects [146]. Thus, by upregulating SNARE activity, and by downregulating cholesterol biosynthesis, H fish may have further mitigated glutamate-driven excitotoxicity.

Another contributor to excitotoxicity is the dysregulation of excess intracellular calcium ([Ca^2+^]_i_) levels among intracellular components [147], which appeared to be more acute in L fish (worksheet e, S12 File). Neurons are typically tolerant of the small [Ca^2+^]_i_ fluctuations [147] required for inter-neuronal signaling [134, 138], but excess [Ca^2+^]_i_ further aggravates glutamate-driven excitotoxicity [147, 148] and can lead to cell death due to electrochemical and energy imbalances (reviewed in [148, 149]). Excess [Ca^2+^]_i_ can enter neurons both through *calcium voltage-gated channels 1a* (*cacna1a*), *cacna1b*, *cacna1c*, and *cacna1e* [134, 150] and through overactive glutamate receptors [151] which were all upregulated in L fish. Another effect of excess [Ca^2+^]_i_ is that it can lead to generation of excessive reactive oxygen species (ROS) production (reviewed in [148, 149]). ROS can also be produced through mitochondrial electron transfer during ATP production (reviewed in [152]), and this process increases with acute temperatures in ectotherms [153, 154]. Evidence of ROS production can be seen in both H and L fish, since both groups upregulated antioxidant genes associated with oxidative stress (worksheets g & h, S12 File). However, H fish upregulated several antioxidant response element (ARE) genes, which are regarded as the primary vertebrate cytoprotective pathway [155]. Regulators of these genes such as *MAF BZIP transcription factor g* (*mafg*) and *k* (*mafk*), *activating transcription factor 4* (*atf4*), and *proto-oncogene c-jun* (*jun*), along with the main co-activators *nuclear factor erythroid 2-like 1* (*nfe2l1*) and *nfe2l2* genes also had greater H fish expression. Together the ARE-regulated genes mount a very powerful response against oxidative stress [156–159]. However, several other ARE-regulated genes showed repressed expression in H fish and potential reasons for these differences are outlined in worksheet h, S12 File. Regardless, H fish appeared to better handle oxidative stress and mitigate neuronal death from excitotoxicity and excess [Ca^2+^]_i_.

### Autophagy

Autophagy appears to be an important component of the response to thermal stress as a large number of interacting genes involved in the recycling of cellular constituents through both macro- and micro- autophagy processes [160–163] showed differential expression between H and L fish (worksheets i & j, S12 File). However, most genes showed elevated expression in H fish, and this was especially evident for two select macroautophagy processes (i.e., aggrephagy and reticulophagy) and their complementary microautophagy processes (i.e., microproteophagy and microreticulophagy, respectively). In particular, the elevation of reticulophagy is characteristic of the UPR due to ER stress (reviewed in [164]). Elevated expression in H fish of multiple cargo carriers such as *sequestosome 1* (*sqstm1*), *toll-interacting protein* (*tollip*), and *optineurin* (*optn*), are also characteristic of aggrephagy and reticulophagy processes linked to increased cell survival [165–167]. H fish also showed increased expression of multiple *endosomal sorting complexes required for transport* (*escrt*) genes. *Escrt* genes are integral to both macro- and microautophagy processes, but subsets of these genes have enhanced expression in microautophagy processes such as microproteophagy and microreticulophagy. Additionally, genes encoding Escrt-III complex proteins that may autonomously regulate endosomal microproteophagy (reviewed in [162]) were elevated in H fish.

H and L fish appear to differ in their propensity to repair and replace lysosomes, which regulate the final stages of autophagic recycling of cellular constituents necessary for cell survival (worksheets i & j, S12 File). The lysosomal membrane can be damaged by protein aggregates [168], which accumulate during thermal stress [169]. Ruptured lysosomes are hazardous to the cell [170], as their contents can trigger cell death (reviewed in [171]; thus, these damaged lysosomes must be either repaired or replaced. Minor damage to the lysosomes can be mitigated through repair pathways, but more extensive damage can require complete replacement of the lysosome [168]. Lysosomal repair mechanisms appeared elevated in H fish for both Escrt-mediated [172], and phosphoinositide-mediated [173] repair pathways (worksheet i, S12 File). However, pathways involved in lysosome regeneration rely on using pre-existing cellular structures as initiators of regeneration differed between H and L fish. For instance, L fish elevated pathways using endosomes or phagosomes as nascent material, as suggested by the greater expression of *phosphoinositide kinase*, *FYVE-Type zinc finger containing* (*pikfyve*) and *mucolipin TRP cation channel 1* (*mcoln1*) regulators in both pathways (reviewed in [174]). Conversely, H fish showed enhancement of the autophagic-source pathway due to the elevated expression of *WASP homolog associated with actin*, *Golgi membranes and microtubules* (*whamm*) and *kinesin family member 5b* (*kif5b*) regulators among others (reviewed in [163, 174]). Lysosomal regeneration in both H and L fish is also supported by the observation that the transcriptional activators (i.e., *transcription factor eb* (*tfeb*) and *transcription factor binding to IGHM enhancer 3* (*tfe3*)) of CLEAR-motif genes that aid in lysosome reconstruction have differential expression of duplicate *tfeb* and *tfe3*.

### Conclusions

We used differential transcriptomic responses in the liver to identify characteristics of high and low thermal tolerance shared in two strains of rainbow trout. Ours is one of the first studies to examine thermal tolerance in this way, and we were able to confirm that the UPR and hsps are indeed important components of the response to thermal stress, but they are not the main factors that differentiate high and low tolerance rainbow trout from one another. Instead, we determined that high tolerance rainbow trout pivot their gene expression to a program of lower gene transcript diversity, while maintaining expression of genes required for the cellular stress response, and of preserving cellular (e.g., by halting the cell cycle, regulating neurotransmitter release, mitigating oxidative stress, removing protein aggregates) and lysosomal (via repair) integrity. Conversely, the increased transcript diversity, elevated levels of cell cycle progression, and increased levels of neuronal glutamate excitotoxicity in L fish were indicators of their inability to regulate metabolic and physiological functions accompanying increased thermal stress. H fish enhanced their thermal tolerance by focusing their efforts on a few key elements that supported their survival, whereas L fishes’ response to thermal stress was less specific, resulting in decreased thermal tolerance. This study builds on existing knowledge of the acute thermal stress response and demonstrates the multifaceted nature of this response as it pertains to multiple biological systems. These findings also have implications to the performance and survival of fish both in the wild and in aquaculture as the result of climate change. The ability to avoid thermal stress may in fact be more challenging in aquaculture as fish will have more limited ability to seek thermal refugia in response to increasing temperatures.

## Supporting information

S1 File Basic RNA-Seq read statistics for the Alma and Lyndon rainbow trout strains. For a more detailed description of all the worksheets in the supplementary files the reader is directed to the DRYAD repository: doi.org/10.5061/dryad.6wwpzgn74.
(XLSX)

S2 File Details on the RNA-Seq DEG results from comparisons between the L vs C, H vs C, and H vs L experimental groups in the Alma strain. For a more detailed description of all the worksheets in the supplementary files the reader is directed to the DRYAD repository: doi.org/10.5061/dryad.6wwpzgn74.
(XLSX)

S3 File Details on the RNA-Seq DEG results from comparisons between the L vs C, H vs C, and H vs L experimental groups in the Lyndon strain. For a more detailed description of all the worksheets in the supplementary files the reader is directed to the DRYAD repository: doi.org/10.5061/dryad.6wwpzgn74.
(XLSX)

S4 File Shared and unique genes among the L vs C, H vs C, and H vs L experimental groups in the Alma and Lyndon strains. For a more detailed description of all the worksheets in the supplementary files the reader is directed to the DRYAD repository: doi.org/10.5061/dryad.6wwpzgn74.
(XLSX)

S5 File Information on gene ontology assignments to shared genes (FDR ≤ 0.01) in the Alma and Lyndon strains. Significant differences (Heterogeneity G-test) between pairwise comparisons in Biological Process (BP), Cellular Component (CC), and Molecular Function (MF) categories are given. For a more detailed description of all the worksheets in the supplementary files the reader is directed to the DRYAD repository: doi.org/10.5061/dryad.6wwpzgn74.
(XLSX)

S6 File Information on the mapped genes in the Alma and Lyndon strains and duplicate gene copies identified. For a more detailed description of all the worksheets in the supplementary files the reader is directed to the DRYAD repository: doi.org/10.5061/dryad.6wwpzgn74.
(XLSX)

S7 File Results from the IPA detailing findings on upstream regulators and pathways from the three pairwise experimental groupings. For a more detailed description of all the worksheets in the supplementary files the reader is directed to the DRYAD repository: doi.org/10.5061/dryad.6wwpzgn74.
(XLSX)

S8 File Graphical representations obtained from the IPA that portray major pathways and genes predicted to be important in the physiological processes discovered among the three pairwise experimental comparisons. For a more detailed description of all the worksheets in the supplementary files the reader is directed to the DRYAD repository: doi.org/10.5061/dryad.6wwpzgn74.
(XLSX)

S9 File IPA pathways observed to be enhanced or suppressed in high tolerance fish in the H vs L experiment in comparison to their status in the H vs C experiment. For a more detailed description of all the worksheets in the supplementary files the reader is directed to the DRYAD repository: doi.org/10.5061/dryad.6wwpzgn74.
(XLSX)

S10 File Identification of functions (IPA BioProfiler) associated with the top 10 regulators identified in both high and low tolerance fish (H vs L comparison). For a more detailed description of all the worksheets in the supplementary files the reader is directed to the DRYAD repository: doi.org/10.5061/dryad.6wwpzgn74.
(XLSX)

S11 File Diseases and functions predicted to increased or decreased in the Alma and Lyndon strains (H vs L analysis), and a reassessment of matches to predicted functional states in the H vs L experiment based upon identified upstream regulators, as well all genes in the H vs L analysis. For a more detailed description of all the worksheets in the supplementary files the reader is directed to the DRYAD repository: doi.org/10.5061/dryad.6wwpzgn74.
(XLSX)

S12 File Listing of key DEG that are known to be important in controlling or regulating transcription & translation, cell cycle, neuronal, antioxidant-stress, and autophagy processes. The file provides information on an expanded list of significant DEG (FDR ≤ 0.1) from the H vs L analysis. For a more detailed description of all the worksheets in the supplementary files the reader is directed to the DRYAD repository: doi.org/10.5061/dryad.6wwpzgn74.
(XLSX)
